# Genome Sequence of *Halomonas* sp. Strain MS1, a Metallophore-Producing, Algal Growth-Promoting Marine Bacterium Isolated from the Green Seaweed Ulva mutabilis (Chlorophyta)

**DOI:** 10.1128/mra.00685-22

**Published:** 2022-10-04

**Authors:** Cristina F. Morales-Reyes, Fatemeh Ghaderiardakani, Thomas Wichard

**Affiliations:** a Friedrich Schiller University Jena, Institute for Inorganic and Analytical Chemistry, Jena, Germany; Montana State University

## Abstract

We report the draft genome sequence of the marine gammaproteobacterium *Halomonas* sp. strain MS1, isolated from the green seaweed Ulva mutabilis (Chlorophyta), which releases metallophores fostering macroalga-bacterium interactions. The 4.6-Mbp sequence, which was obtained using PacBio technology, harbors 4,166 predicted coding sequences, including gene clusters for siderophore production.

## ANNOUNCEMENT

*Halomonas* sp. strain MS1 was initially isolated from the green seaweed Ulva mutabilis (Chlorophyta) and taxonomically classified by 16S rRNA analysis (GenBank accession number EU359908.1) (1). *Ulva* is used as a model organism for macroalga-bacteria interactions, specifically bacteria-induced algal growth and morphogenesis (2). It was hypothesized that those *Ulva*-bacteria interactions are fostered through bacterial siderophore production, which promotes iron uptake by the macroalgae (3). Preliminary studies demonstrated that *Halomonas* sp. strain MS1 releases metal-binding ligands (3, 4), also named metallophores (5, 6), as demonstrated for other bacteria of the genus *Halomonas* (7, 8). The genome of *Halomonas* sp. strain MS1 was thus sequenced to study organic ligands as potential algal growth- and morphogenesis-promoting factors (AGMPFs) in *Ulva* development (9) through their contributions to metal acquisition and detoxification (8–10). To compensate for low iron bioavailability, bacteria frequently produce an excess of siderophores (11). Moreover, metallophores released by *Halomonas* species might serve as a “public good” and in turn as a carbon source (12) in the chemosphere of *Ulva* species, suggesting a mutualistic exchange according to the “carbon-for-iron mutualism” hypothesis (13, 14).

The *Halomonas* species was cultivated for 4 days in a marine broth medium (Carl Roth, Germany) in polycarbonate flasks at 20 ± 1°C in natural light with orbital shaking (1). From laboratory cryostocks following the initial isolation, the genomic DNA was extracted according to the cetyltrimethylammonium bromide protocol (15). The strain is available on request from the culture collection of the corresponding author.

The DNA sample was sheared in Covaris g-TUBEs, resulting in fragments with a target size of 20 kb. A Pacific Biosciences (PacBio) SMRTbell Express library was prepared according to the manufacturer's instructions using AMPure PB beads for size selection. Microsynth AG (Switzerland) performed library barcoding and pooling/multiplexing, library quality control (Femto Pulse analysis and BluePippin size selection), genome sequencing on a PacBio Sequel sequencer, and subsequent assembly. Upon subread generation (PacBio single-molecule real-time [SMRT] Link v.10.1) and extraction (SAMtools v.1.14), Filtlong v.0.2.1 was used to filter subreads smaller than 1,000 bp. *De novo* assembly was carried out by using the hierarchical pipeline Canu v.2.2, including error correction and detection of circularity (16). The read-trimming task used overlapping reads to decide what regions should be trimmed. Default parameters were applied for all software packages.

Sequencing with a SMRT Cell generated a total of 2,493,225,820 bases and 314,515 nucleotide sequences (read *N*_50_, 9,636 bp), resulting in 1 contig consisting of 4.6 Mbp, with a GC content of 53% and an average reference coverage of 538×.

The assembled *Halomonas* sp. strain MS1 genome was annotated via the NCBI Prokaryotic Genome Annotation Pipeline (PGAP) using the best-placed reference protein set (GeneMarkS-2+) v.6.1. This resulted in 4,166 predicted coding sequences, with 61 tRNAs, six 5S rRNAs, six 16S rRNAs, and six 23S rRNAs. AntiSMASH v.5.0 (17) analysis revealed several secondary metabolic biosynthetic gene clusters, including those for metallophore production. In addition, RAST Server v.2.0 (18) analysis revealed 19 iron acquisition, metabolism, and siderophore features. The findings are consistent with previous reports regarding *Halomonas* species genomes (19–21).

### Data availability.

This whole-genome shotgun project has been deposited in GenBank under accession number CP098926, with BioProject accession number PRJNA828511 and SRA accession number SRR19647641.
